# Photovoltaic Power Prediction Based on Hybrid Deep Learning Networks and Meteorological Data

**DOI:** 10.3390/s24051593

**Published:** 2024-02-29

**Authors:** Wei Guo, Li Xu, Tian Wang, Danyang Zhao, Xujing Tang

**Affiliations:** 1 School of Naval Architecture, Ocean and Energy Power Engineering, Wuhan University of Technology, Wuhan 430063, China; gw94@whut.edu.cn (W.G.);; 2 State Key Laboratory of Maritime Technology and Safety, Wuhan University of Technology, Wuhan 430063, China

**Keywords:** photovoltaic power forecasting, quantile regression, probabilistic forecasting, deep learning hybrid model

## Abstract

Conventional point prediction methods encounter challenges in accurately capturing the inherent uncertainty associated with photovoltaic power due to its stochastic and volatile nature. To address this challenge, we developed a robust prediction model called QRKDDN (quantile regression and kernel density estimation deep learning network) by leveraging historical meteorological data in conjunction with photovoltaic power data. Our aim is to enhance the accuracy of deterministic predictions, interval predictions, and probabilistic predictions by incorporating quantile regression (QR) and kernel density estimation (KDE) techniques. The proposed method utilizes the Pearson correlation coefficient for selecting relevant meteorological factors, employs a Gaussian Mixture Model (GMM) for clustering similar days, and constructs a deep learning prediction model based on a convolutional neural network (CNN) combined with a bidirectional gated recurrent unit (BiGRU) and attention mechanism. The experimental results obtained using the dataset from the Australian DKASC Research Centre unequivocally demonstrate the exceptional performance of QRKDDN in deterministic, interval, and probabilistic predictions for photovoltaic (PV) power generation. The effectiveness of QRKDDN was further validated through ablation experiments and comparisons with classical machine learning models.

## 1. Introduction

### 1.1. Problem Statement

In recent years, the rapid development of renewable energy, particularly photovoltaic (PV), has led to a gradual increase in its share within the installed capacity of the power system. However, as PV power penetration rates rise, the inherent randomness and volatility associated with it may have an impact on the main power grid [1]. Therefore, the accurate prediction of PV power generation is crucial for enabling the power dispatch department to formulate a rational power generation plan that supports frequency and voltage regulation within the power grid, ensuring both security and economic efficiency in the electricity supply [2].

### 1.2. Literature Survey

PV power prediction is categorized based on the prediction process, spatial scale, form, and method [3]. In recent years, deep learning methods have garnered significant attention from researchers due to their exceptional feature extraction and transformation capabilities, leading to remarkable achievements in PV power prediction [4]. Long short-term memory (LSTM), as a classical deep learning approach, with its unique architecture facilitating the transfer of available information from previous states to the current state through memory units, is well suited for PV power forecasting [5,6,7]. The aforementioned studies did not succeed in enhancing the forecasting accuracy through improvements to the LSTM structure. However, a pioneering study [8] introduced LSTM into an independent PV day-ahead power prediction model and proposed a correction method that considers the correlation among different PV power generation modes, thereby improving the predictive accuracy of the LSTM model. To address the issue of slow convergence in LSTM [9,10], this study combined a gated recurrent unit (GRU) with weather forecast data to predict horizontal irradiance for a 24-h period. The results demonstrate that GRU outperforms LSTM in terms of prediction error and training time. Compared to alternative deep learning methodologies, CNN networks enable the efficient extraction of usable features from extensive training data and employ multiple convolutional kernels as feature extractors to enhance the performance of feature extraction. These techniques have been successfully applied in various time series prediction domains, including wind speed prediction, solar irradiation prediction, and photovoltaic power prediction [11,12,13]. In contrast to the limitations of single model predictions, the integration of multiple models allows for the leveraging of their respective strengths and the effective harnessing of information from PV power data and meteorological data series, thereby significantly improving prediction accuracy. The fusion of CNN and LSTM in [14] demonstrates that the combined model outperforms individual models based on a real-world Moroccan dataset. In [15], the convolutional long- and short-term memory model (CLSTM) was optimized using the enhanced sparrow search algorithm (SSA). Comparative experiments conducted on real operational data from a photovoltaic power plant in northern China demonstrated that the PV output prediction accuracy of the CLSTM hybrid neural network, based on optimized parameters obtained through improved SSA, significantly outperforms that of individual neural networks such as back propagation (BP), CNN, and LSTM. Furthermore, it surpasses the prediction accuracy of an unoptimized CLSTM hybrid neural network. In another study [16], a TSF-CGANs algorithm was proposed by integrating conditional generative adversarial networks (CGANs) with CNNs and bidirectional long short-term memories (BiLSTM)s. The results obtained from real data predictions indicate that the time series forecasting based on the CGANs (TSF-CGANs) algorithm exhibits superior prediction accuracy compared to traditional single models. Additionally, ref. [17] introduces a similar day model clustering fusion CNN-Informer for PV power prediction, which utilizes CNN for feature extraction and combines its outputs with Informer model inputs. By leveraging information source modeling techniques to establish temporal feature correlations among historical data, this approach achieves accurate PV power predictions.

The point prediction method is a deterministic approach, but it fails to capture the probability distribution and fluctuation range of the prediction results. In complex weather conditions, photovoltaic power generation exhibits significant fluctuations within short periods, thereby compromising the accuracy of the point prediction method and posing challenges for maintaining a stable and secure power grid [18,19,20]. Probabilistic density prediction, on the other hand, offers a more comprehensive forecasting technique by effectively representing uncertainty as a probability distribution centered around the predicted value. This is achieved through QR and KDE, enabling operators to obtain prediction intervals in terms of a probability density function (PDF) for improved decision making [21]. Currently, the research on the probabilistic prediction of PV power is in its nascent stage. With a focus on ensuring accurate point predictions, current research aims to establish machine learning-based models such as QR [22], Gaussian process regression (GPR) [23], and KDE [24] to obtain prediction intervals and probabilistic density functions for PV power predictions under fixed confidence conditions. The research [25] developed a PV power prediction interval model based on linear programming, employing an extreme learning machine and QR method. The effectiveness of the method and the higher computational efficiency of the model were verified through a numerical study using Danish PV data, enabling the accurate quantification of the variability and uncertainty in electricity generation from PV systems. The proposed PV power probabilistic prediction method [26] is based on the dynamic weighting method, k-nearest neighbor (KNN) algorithm, and quantile regression neural network (QRNN). Its validity was confirmed through validation using the IEEE Working Group on Energy Forecasting (IEEE WGEF) data, thus establishing its credibility. In another study [27], Bayesian bootstrapping was applied to three probabilistic prediction models: linear quantile regression, the gradient augmented regression tree, and the quantile regression neural network. Sample bootstrap distributions were computed to predict power quartiles and conduct probabilistic prediction tests on two real PV power generation datasets: the HEIG-VD ReIne Lab and Global Energy Forecasting Competition 2014 (GEFCOM2014). The effectiveness of this approach was demonstrated. The authors of [28] proposed a PV power prediction model based on various meteorological data, including cloudiness and visibility. They developed a hybrid prediction method that combines QR with a coupled input forgetting gate (CIFG) network to predict the conditional quartiles of PV output power. Additionally, they employed a KDE method to estimate the probability density function of PV output. Probabilistic forecasting has also been explored in other research domains, such as wind power forecasting and load forecasting. The authors of [29] introduced a wind speed interval prediction approach using variational modal decomposition (VMD), phase space reconstruction (PSR), a whale optimization algorithm (WOA), QR, and gated recurrent unit networks (GRU). They established a PSR-IWOA-QRGRU model for wind speed interval prediction by superimposing the predictions from different components. A hybrid generalized forecasting framework was developed by a study [30], which proposed a probabilistic wind speed prediction method in the form of point estimation and interval prediction. This approach combines empirical wavelet transform with neural network-based QR to enhance the generalization and stability of probabilistic forecasting. In addition, ref. [31] introduced a probability density forecasting approach based on Yeo-Johnson transformed QR of Gaussian kernel functions, combining empirical bandwidth-based Gaussian kernel density estimation with Yeo-Johnson transformed QR for short-term electricity load probability density forecasting. The performance of the presented model was validated using one-hour historical load data for August, summer, and December, winter, 2014 in Ottawa, Canada. In a study [32], a QRNN probabilistic load forecasting model considering both temperature uncertainty and load variations was proposed, an innovative quantile regression neural network with parameter embedding was built to capture the load variations, and temperature forecasts were generated in a probabilistic manner using temperature scenario-based techniques, and the results show that the proposed method outperforms the commonly used benchmark models.

### 1.3. Motivation of the Study

The accurate estimation of the fluctuation interval in output power is essential for grid dispatching due to the intermittent and fluctuating nature of the photovoltaic power supply. Deterministic point prediction fails to quantitatively describe the uncertainty associated with PV power, whereas probability interval prediction can provide a range of fluctuations in predicted power, along with upper and lower bounds at a certain confidence level. Simultaneously, predicting the probability distribution and confidence interval of the photovoltaic power output enhances the reliability of the photovoltaic power station output, guides reactive power planning in distribution networks, facilitates real-time power operation planning, and effectively promotes renewable energy consumption. Moreover, the existing probabilistic interval models exhibit inadequate reliability and sensitivity, particularly when confronted with significant fluctuations in PV power. Consequently, a single traditional model alone cannot achieve accurate predictions. To address this issue, we propose a hybrid approach for PV power prediction in this paper. Our model integrates CNN, BiGRU, and attention mechanisms to enable probabilistic forecasting encompassing point estimation, interval prediction, and probability density estimation.

### 1.4. Research Content

The core research content and innovation of this article are as follows:

(1) In this paper, the QRKDDN short-term PV power prediction model is proposed by fusing the QR and KDE methods and combining CNN, BiGRU, and attention mechanisms.
(2) The proposed probabilistic interval prediction model is validated through deterministic, interval, and probabilistic predictions to provide valuable insights for quantifying the uncertainty associated with future PV power.
(3) The significance of data preprocessing in short-term PV power forecasting is investigated in this study. The Pearson correlation coefficient is employed to perform correlation analysis on the variables in the dataset, and the utilization of multivariate inputs enables the model to effectively capture interdependencies between variables, thereby enhancing the accuracy of PV power prediction. Additionally, the clustering of PV data on similar days is conducted using the GMM method, and comparative experiments demonstrate that this approach significantly improves prediction precision.

The subsequent sections of this paper are organized as follows: Section 2 provides an overview of the relevant methodologies employed in this study. Section 3 demonstrates the efficacy of the proposed approach through illustrative examples. Finally, Section 5 presents a comprehensive conclusion.

## 2. Methods

### 2.1. Gaussian Mixture Model

The Gaussian Mixture Model is a probabilistic clustering method that effectively captures attribute correlation and dependency, unlike traditional approaches such as hierarchical clustering and fuzzy clustering, which heavily rely on initial conditions and distance measures [33]. The GMM algorithm operates by assigning clusters solely based on probability theory. The principle of the GMM algorithm is briefly described as follows:

Assuming K represents the number of GMM clusters, the mean $\mu_{0}$, covariance $\sum_{k}$, and weights $\omega_{0}$ of the parameters are randomly initialized. The expectation-maximization (EM) algorithm comprises an E-step and an M-step. In the E-step, the probability that each sample point $z_{i}$ belongs to the kth distribution is calculated using the following expression:

(1) $$\gamma_{k}\left(z_{i}\right)=\frac{\omega_{k}N(z_{i}|\mu_{k},\sum_{k})}{\sum_{k=1}^{k}\omega_{k}N(z_{i}|\mu_{k},\sum_{k})}$$

where $N(z_{i}|\mu_{k},\sum_{k})$ is the Gaussian probability density function, and $\mu_{k}$, $\sum_{k}\;$, and $\omega_{k}$ are the mean, covariance, and weight of the *k*th distribution, respectively.

The parameters of each distribution are solved for using the M-step and updated with the expression

(2) $$\mu_{k}=\frac{\sum_{i=1}^{N}\gamma_{k}\left(z_{i}\right)x_{i}}{\sum_{i=1}^{N}\gamma_{k}\left(z_{i}\right)}$$

(3) $$\sum_{k}=\frac{\sum_{i=1}^{N}\gamma_{k}\left(z_{i}\right)\left(z_{i}-\mu_{k}\right){\left(z_{i}-\mu_{k}\right)}^{T}}{\sum_{i=1}^{N}\gamma_{k}\left(z_{i}\right)}$$

(4) $$\omega_{k}=\frac{1}{N}\sum_{i=1}^{N}\gamma_{k}\left(z_{i}\right)$$

The aforementioned steps were iteratively performed until the parameters reached convergence. Subsequently, the sample points were effectively clustered by means of the resulting GMM.

The clustered feature vectors were obtained by utilizing the mean and standard deviation as feature metrics, while transforming the five meteorological factors exhibiting strong correlation and historical PV power into daily feature metrics. The resulting clustered feature vector is denoted as $X_{j}=\left[X_{j,1},X_{j,2},\cdots ,X_{j,10}\right](j=1,2,\cdots ,N)$. According to the Bayesian information criterion (BIC), the optimal number of clusters for GMM is calculated as 3, enabling the classification of PV power fluctuation characteristics into three distinct weather types: sunny, cloudy, and rainy days. Consequently, a set of samples representing similar PV power patterns on different days was established.

### 2.2. Multivariate Correlation Analysis

The power generation efficiency of photovoltaic systems is influenced by various environmental variables to varying degrees. Employing the Pearson correlation coefficient method for meteorological factor analysis and selecting environmental variables with higher correlation coefficients as prediction inputs can enhance the accuracy of prediction models [16]. The Pearson correlation coefficient is calculated as follows:

(5) $$\rho_{x,y}=\frac{\sum_{i=1}^{n}\left(x_{i}-\bar{x}\right)\left(y_{i}-\bar{y}\right)}{\sqrt{\sum_{i=1}^{n}{\left(x_{i}-\bar{x}\right)}^{2}}\sqrt{\sum_{i=1}^{n}{\left(y_{i}-\bar{y}\right)}^{2}}}$$

where $\bar{x}$ and $\bar{y}$ represent the respective mean values of variables $x_{i}$ and $y_{i}$, and a positive correlation coefficient $\rho_{x,y}$ indicates a direct relationship between the two variables. Conversely, a negative correlation coefficient suggests an inverse relationship, with values closer to 0 indicating weaker degrees of correlation.

### 2.3. Quantile Regression

The conditional quantile relationship between the independent variable $X=\left[x_{1},x_{2},\cdots ,x_{n}\right]$ and the dependent variable $Y=\left[y_{1},y_{2},\cdots ,y_{n}\right]$ can be estimated using the QR method. Unlike traditional regression techniques, which rely on assumptions about the error distribution, QR directly models the error distribution function. Therefore, it does not impose any restrictive assumptions regarding datasets or prediction error normality [34]. The corresponding formula for QR is as follows:

(6) $$Q_{y_{t}}\left(\tau |x_{t}\right)=f\left[x_{t},\beta \left(\tau \right)\right],t=1,2,\cdot \cdot \cdot ,n$$

where $Q_{y_{t}}\left(\tau |x_{t}\right)$ is the conditional quantile of the dependent variable, where the value of τ ranges from 0 to 1. $\beta \left(\tau \right)$ is the regression coefficient, the estimate of which is calculated by the formula

(7) $$\hat{\beta }\left(\tau \right)=argmin\sum_{t=1}^{n}\psi_{\tau }\left[y_{t}-x_{t}\beta \left(\tau \right)\right]$$

(8) $$\psi_{\tau }\left(u\right)=\left\{\begin{array}{c} \tau u,u\geq 0\\ \left(\tau -1\right)u,u<0 \end{array}\right.$$

where $\psi_{\tau }\left(u\right)$ is an asymmetric function.

The conditional quantile of the dependent variable $y_{t}$ is

(9) $${\hat{Q}}_{y_{t}}\left(\tau |x_{t}\right)=x_{t}\hat{\beta }\left(\tau \right)$$

### 2.4. Kernel Density Estimate

Similar to QR, KDE is a non-parametric method that enables the direct calculation of the probability density for predicting PV power values without making distributional assumptions. In this study, we employed the cosine kernel function as the KDE technique [35]. The formula for KDE computation is presented as follows:

(10) $${\hat{f}}_{d}\left(x\right)=\frac{1}{Nd}\sum_{i=1}^{N}K\left(\frac{T_{i}-x}{d}\right)$$

where d is the bandwidth, and d>0. The variable N represents the total count of quartiles, while *T* denotes the dataset comprising conditional quartiles. K(α) refers to the cosine kernel function, and its formula is presented as follows:

(11) $$K\left(\alpha \right)=\left\{\begin{array}{c} \frac{\pi }{4}\cos\frac{\pi }{2}\alpha ,\alpha \in \left[-1,1\right]\\ 0,\alpha \notin \left[-1,1\right] \end{array}\right.$$

The model performance is optimized by employing grid search methods, which systematically explore various combinations of parameters. In this study, a cross-validation-based grid search approach was employed to select the bandwidth parameter relevant to the research [36].

### 2.5. Convolutional Neural Network

Due to space limitations, this article provides a brief description of the basic model structure. The CNN network effectively leverages the correlation between historical weather data of PV power plants and PV power generation for extracting significant features, which can be mathematically represented by Equations (12) and (13) [37].

(12) $$C_{i}=f\left(C_{i-1}⊗W_{i}+b_{i}\right)$$

(13) $$C_{0}=I$$

where $C_{i}$ and $C_{i-1}$ are the feature outputs of layers i and i−1; ⊗ is the convolution operation; $b_{i}$ denotes the offset of layer i; and the original input $C_{0}$ corresponds to I.

**Figure 1:**
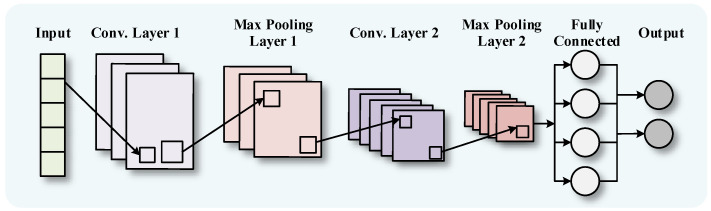
CNN network structure.

The classical CNN architecture is depicted in Figure 1 [38].

### 2.6. BiGRU Model

LSTM networks possess the capability to acquire correlation information between long- and short-term sequential data, while GRU, as a variant of LSTM with reduced parameters, exhibits a faster convergence rate. In contrast to LSTM, GRU replaces the input and forgetting gates with update gates [39]. The computation of the GRU hidden layer unit $h_{t}$ can be derived from Equations (14)–(17).

(14) $$r_{t}=\sigma \left(W_{r}x_{t}+U_{r}h_{t-1}\right)$$

(15) $$z_{t}=\sigma \left(W_{z}x_{t}+U_{z}h_{t-1}\right)$$

(16) $${\tilde{h}}_{t}=\tanh\left(r_{t}\circ Uh_{t-1}+Wx_{t}\right)$$

(17) $$h_{t}=\left(1-z_{t}\right)\circ {\tilde{h}}_{t}+z_{t}\circ h_{t-1}$$

where $z_{t}$ and $r_{t}$ represent the updated and reset gates, respectively; σ denotes the sigmoid function; and $W_{r}$, $U_{r}$, $W_{z}$, $U_{z}$, W, and U are matrices of training parameters. $r_{t}$ is the reset gate, $h_{t-1}$ is the hidden layer neuron output of the previous moment, $x_{t}$ is the input of the present moment, and W and U denote the matrices of the training parameters, which collectively determine ${\tilde{h}}_{t}$, the candidate activation state of the current moment. Additionally, $z_{t}\circ h_{t-1}$ signifies the composite relationship between $z_{t}$ and $h_{t-1}$.

**Figure 2:**
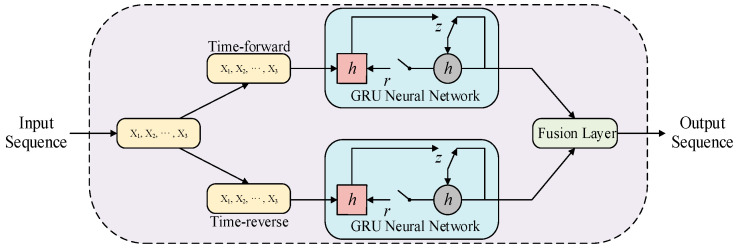
The structure of the BiGRU network.

The flow of information in a unidirectional neural network is typically sequential, propagating from front to back. However, the photovoltaic power at any given moment exhibits correlations with both past and future periods. To capture the deep features of PV power data, the BiGRU network integrates historical and future information seamlessly. Figure 2 illustrates the structure of the BiGRU model [40].

### 2.7. Attention Mechanism

**Figure 3:**
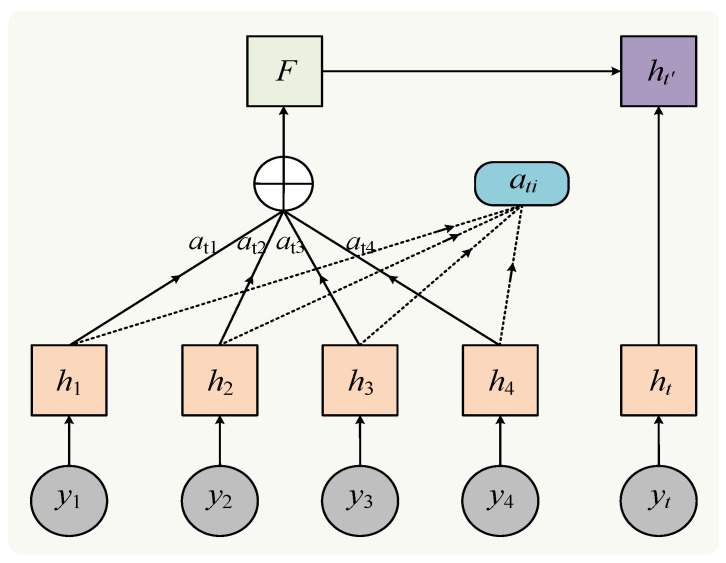
Attention unit structure.

The attention mechanism is rooted in the modeling of attentional characteristics observed in the human brain, which enhances information processing efficiency by allocating differential weights [41]. Figure 3 illustrates the structure of the attention unit. The expression for the attention mechanism is presented below:

(18) $$e_{i}=u\tanh\left(wh_{i}+b\right)$$

(19) $$\alpha_{i}=\frac{\exp\left(e_{i}\right)}{\sum_{i}\exp\left(e_{i}\right)}$$

(20) $$C=\sum_{i}\alpha_{i}h_{i}$$

The attention score at moment i is denoted by $e_{i}$, where u and w are the weighting coefficients, b represents the bias coefficient, $a_{i}$ signifies the feature weights, and C represents the output of the attention layer at time i.

### 2.8. Structure of the QRKDDN Model

**Figure 4:**
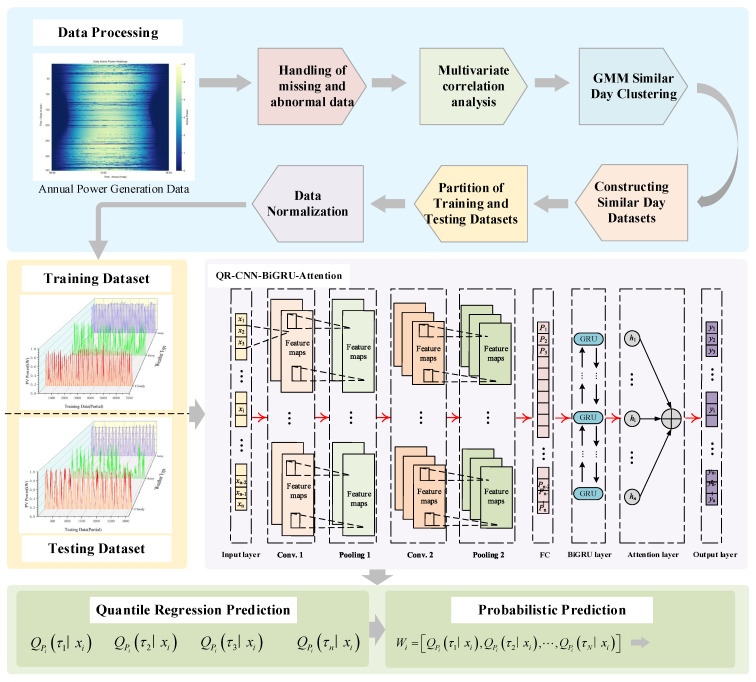
The proposed QRKDDN.

The structure of the QRKDDN model proposed in this paper is shown in Figure 4. The following provides a concise elucidation of the principles and procedures involved in forecasting. After preprocessing the historical data of photovoltaic power, the Pearson correlation coefficient method is employed to select the correlations among meteorological variables. The GMM algorithm is employed to cluster historical PV power data from similar days, followed by the division of training and test sets, and normalization prior to inputting into the prediction model. The QRKDDN model consists of a CNN layer, bidirectional BiGRU layer, and attention layer. The CNN layer exhibits strong local feature extraction capabilities, effectively tracking the actual PV power prediction value and reducing uncertainty during periods of sharp power fluctuations. The bidirectional BiGRU neural network captures long-term dependent relationships within sequences, enabling it to capture changes in internal information features, which are then inputted into the attention mechanism layer. This attention mechanism dynamically assigns weights to output vectors based on weight distribution principles, calculating corresponding probabilities for different feature vectors. Through constant updates and iterations of optimal weight parameter matrices, high-precision prediction of photovoltaic power is achieved. Prediction intervals are generated using the QR algorithm, while probability prediction results are obtained through KDE methods.

## 3. Case Study

### 3.1. Data Description

**Figure 5:**
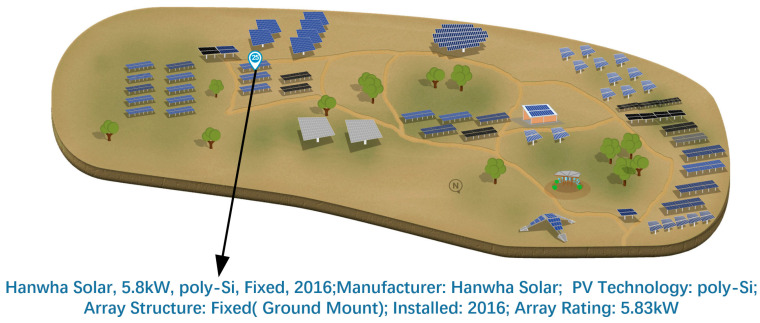
The map of the system.

In this study, the Desert Knowledge Australia Solar Centre (DKASC) Hanwha Solar dataset was selected as the research subject. Specific information about this PV power plant is presented in Figure 5 [42]. The original data used for analysis encompass the output power of the PV generation system and meteorological data collected through an array of sensors from 1 January to 31 December 2020. The weather data comprise crucial meteorological variables, including temperature, relative humidity, radiation data, and rainfall. To ensure the accuracy of the results, only data collected between 6:00 and 19:00 each day were retained for analysis due to the negligible power output during the morning and evening hours. The raw resolution of the dataset was set at five-minute intervals, with a total of 163 sampling points throughout the day. A training-to-test ratio of 7–3 was employed.

**Figure 6:**
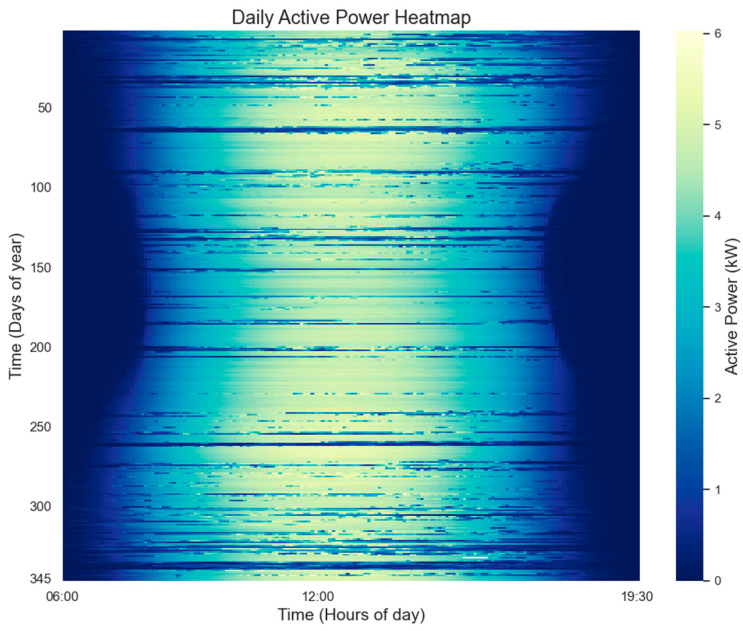
Thermogram of photovoltaic power generation for the whole year.

Due to equipment failure or maintenance, potential data loss may occur, necessitating data processing as a preliminary step. In cases where the daily sampling data exhibited a continuous absence of ≤3 points, interpolation was performed using the upper and lower mean padding method; however, if there were more than 3 missing values or consecutively missing points in the daily sampling data, the entire day’s dataset was excluded from analysis. Following the interpolation process for handling missing values, a total of 345 days’ worth of data were retained throughout the year. Outliers were identified using the box plot method and replaced by taking an average between adjacent non-outlier data points before and after each outlier occurrence. The processed thermogram illustrating PV power over the course of one year is presented in Figure 6.

The utilization of normalized data in prediction aims to mitigate the influence of data dimensionality on prediction outcomes and reduce training time. Nevertheless, it is crucial that the final outcome represents the predicted photovoltaic power generation value, necessitating a comparison with actual power generation for evaluating predictive performance. Consequently, reverse normalization becomes imperative. The formulas for both normalization and reverse normalization are presented as follows:

(21) $$x_{t}^{\prime }=\frac{\left(x_{t}-x_{min}\right)}{\left(x_{max}-x_{min}\right)}$$

(22) $$x_{t}=\left(x_{max}-x_{min}\right)x_{t}^{\prime }+x_{min}$$

The variable $x_{t}$ represents the sample value, $x_{max}$ and $x_{min}$ denote the sample maximum and minimum values, respectively, and $x_{t}^{\prime }$ is the sample normalized value.

### 3.2. Evaluation Indicators

In this paper, root mean squared error (RMSE) and goodness of fit R^2^ are selected as the evaluation metrics for point prediction, with the following formulas:

(23) $$e_{RMSE}=\sqrt{\frac{1}{n}\sum_{i=1}^{n}{\left(y_{i}-{\hat{y}}_{l}\right)}^{2}}$$

(24) $$R^{2}=1-\frac{\sum_{i=1}^{n}{\left(y_{i}-{\hat{y}}_{i}\right)}^{2}}{\sum_{i=1}^{n}{\left(y_{i}-\sum_{i=1}^{n}\frac{y_{i}}{n}\right)}^{2}}\times 100\%$$

where $y_{i}$ and ${\hat{y}}_{i}$ represent the true power value and the model-predicted value at moment i, respectively; n denotes the number of test samples. A lower RMSE indicates higher prediction accuracy, while a value of $R^{2}$ closer to 1 suggests more accurate predictions.

Interval evaluation metrics such as PICP (prediction interval coverage probability) and PINAW (prediction interval normalized average width) were employed. The PICP value represents the probability that an observation falls within the upper and lower bounds of the prediction interval at a given confidence level, with higher values indicating better prediction accuracy. When comparing equal PICP values, smaller PINAW values indicate superior predictions. The formula is as follows:

(25) $$I_{PICP}=\frac{1}{N}\sum_{n=1}^{N}S_{n}$$

(26) $$I_{PINAW}=\frac{1}{NE}\sum_{i=1}^{N}\left(P_{upi}-P_{downi}\right)$$

where $I_{PICP}$ represents the coverage value of the prediction interval, and $I_{PINAW}$ denotes the average width value of the prediction interval. $S_{n}$ is a binary variable, taking a value of 1 when the observation falls within the prediction interval and 0 otherwise. E represents the range between the maximum and minimum values of the observation, while $P_{upi}$ and $P_{downi}$ represent, respectively, the upper and lower bounds of the prediction interval.

The continuous ranked probability score (CRPS) is commonly employed to assess probabilistic predictions’ accuracy, with smaller CRPS values indicating higher accuracy. The formula for CRPS is as follows:

(27) $$P_{CRPS}=\frac{1}{N}\sum_{i=1}^{N}\int_{-\infty }^{+\infty }{\left(F\left(P_{pi}\right)-H\left(P_{pi}-P_{ri}\right)\right)}^{2}dP_{pi}$$

(28) $$F\left(P_{pi}\right)=\int_{-\infty }^{P_{pi}}p\left(x\right)dx$$

(29) $$H\left(P_{pi}-P_{ri}\right)=\left\{\begin{array}{ll} 0, & P_{pi}<P_{ri}\\ 1, & P_{pi}\geq P_{ri} \end{array}\right.$$

where P(x) represents the probability density function, $F\left(P_{pi}\right)$ denotes the cumulative distribution function of $P_{pi}$, and $H\left(P_{pi}-P_{ri}\right)$ corresponds to the step function.

### 3.3. Feature Selection

**Figure 7:**
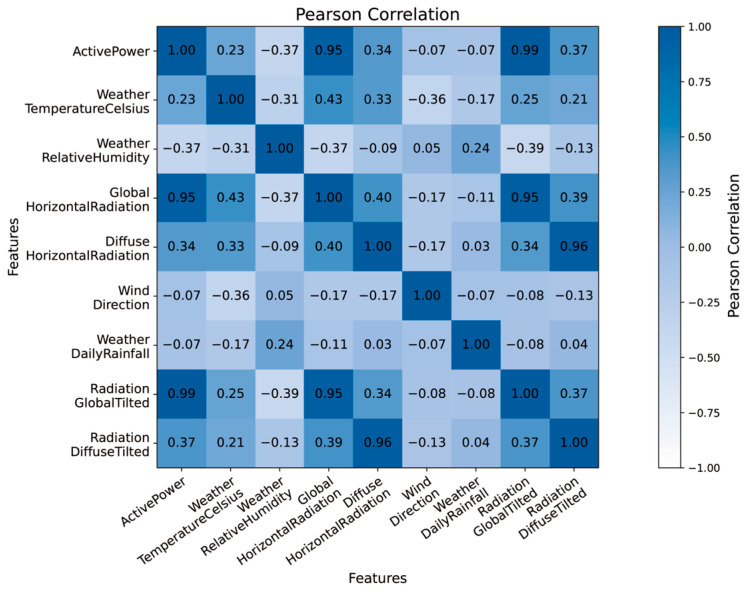
Pearson correlation coefficient applied to photovoltaic power dataset.

The Pearson correlation coefficient heatmap offers a more intuitive depiction of the interdependence among variables, as illustrated in Figure 7. Based on the level of correlation, we selected global tilted radiation, global horizontal radiation, diffuse tilted radiation, weather relative humidity, and diffuse horizontal radiation as input variables for the predictive model.

### 3.4. Similar Day Clustering

**Figure 8:**
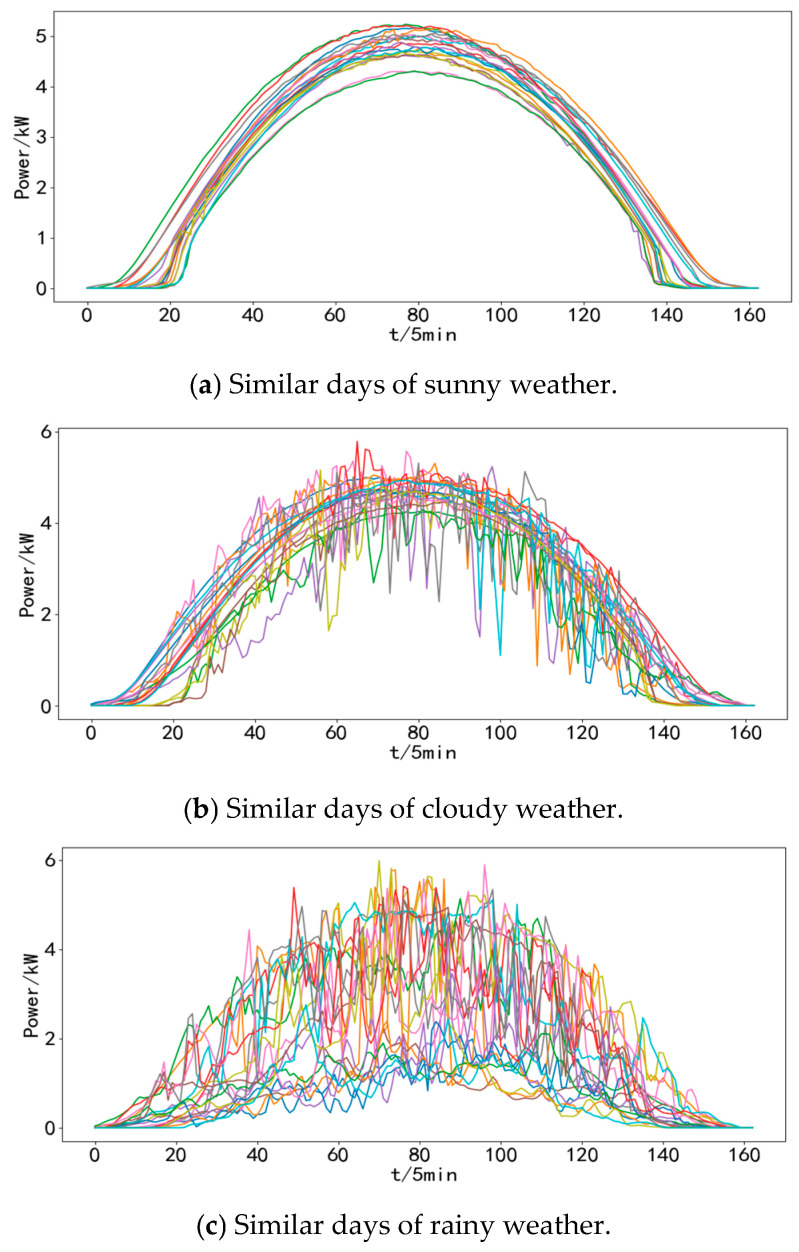
Similar day clustering results (20 random days).

The GMM clustering method was employed to identify similar days for the raw PV power samples, and the dataset of 345 days in a year was categorized into three types of similar day samples: sunny, cloudy, and rainy. Specifically, there were 172 sunny days, 112 cloudy days, and 61 rainy days. The clustering outcomes for different weather conditions are illustrated in Figure 8 (only data for a randomly selected subset of 20 days are presented).

### 3.5. Parameter Settings

The model structure and parameter settings are presented in Table 1. After multiple rounds of experimental testing and optimization, the QRKDDN parameter is set to achieve optimal performance. To ensure experimental comparability, the structural parameters and experimental settings of the comparison models (QR-GRU, QR-BiGRU, QR-BiGRU-Attention, QR-CNN-BiGRU, and QR-CNN-BiLSTM-Attention) adhere to the standards defined in QRKDDN. Due to space limitations within this paper, a detailed description of the comparative model’s structure is omitted; however, it can be found in the reference, along with its schematic diagram.

All the experiments in the article were conducted in a computing environment based on an Intel(R) Core(TM) i7-11800H (2.30 GHz) 16 GB RAM (Intel, Santa Clara, CA, USA) and Windows 64-bit operating system (Microsoft, Redmond, DC, USA), and the proposed main algorithmic model is built by frameworks such as Tensorflow 2.12, Keras, etc., and is written in Python 3.9.

## 4. Results

To showcase the advancements of QRKDDN in the short-term interval prediction and probabilistic forecasting of PV power, a comparative analysis was conducted between the prediction results obtained from the QRKDDN model and those derived from a comparative model across three distinct weather types. For visualization and analysis purposes, one day per weather type was randomly selected. Meanwhile, three specific time points during each day (9:00 a.m., 12:00 noon, and 5:00 p.m.) were chosen to plot the probabilistic prediction outcomes. The predictions were averaged over 10 runs of the models, with a confidence level set at 95%.

### 4.1. Sunny

**Figure 9:**
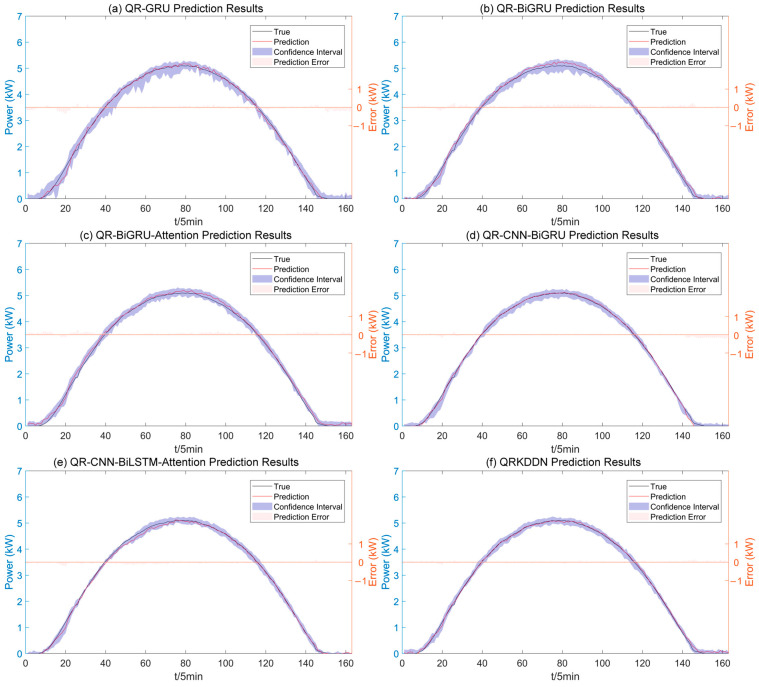
Interval prediction results for sunny weather.

The prediction results of the QRKDDN model and the comparison model under sunny conditions are illustrated in Figure 9.

**Figure 10:**
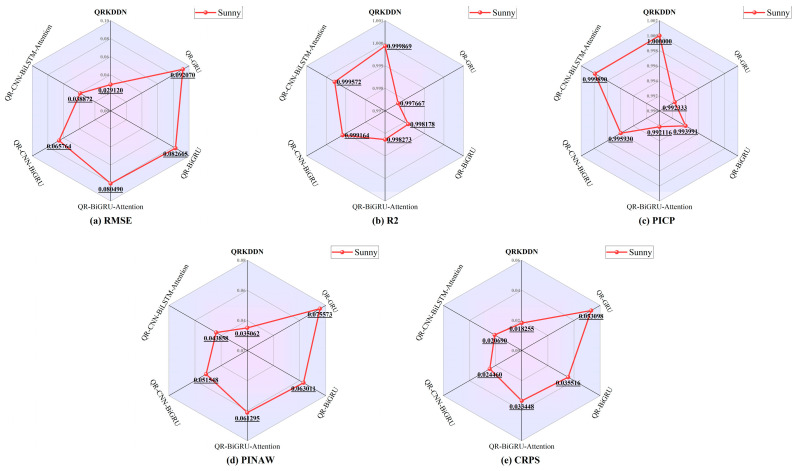
Radar chart of predictive evaluation indicators of sunny weather.

As depicted in Figure 9, the QRKDDN model exhibits the most favorable prediction interval width. To facilitate visual comparison of the predictive performance among models, a radar plot of the evaluation metrics for the sunny day dataset is presented in Figure 10.

According to Figure 10, QRKDDN exhibits the smallest RMSE of 0.029120 and the highest $R^{2}$ value of 0.999869 under sunny weather conditions. The prediction interval coverage achieves a perfect score of 100%, while simultaneously demonstrating the narrowest prediction interval width with a value of 0.035062 for PINAW. Notably, the QRKDDN model outperforms other models in terms of its optimum CRPS, surpassing the QR-GRU model by a margin of 65.62%, being 48.60% lower than the QR-BiGRU model, exhibiting a reduction of 45.42% compared to the QR-BiGRU-Attention model, showcasing an improvement of 25.37% relative to the QR-CNN-BiGRU model, and achieving an enhancement of 11.77% when compared to the QR-CNN-BiLSTM-Attention model.

**Figure 11:**
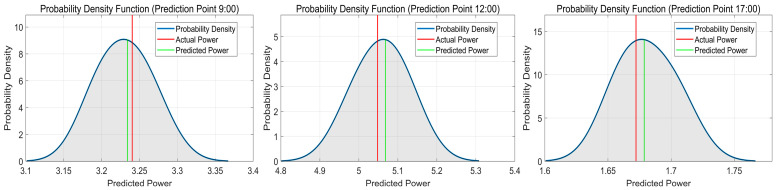
Probabilistic prediction results of QRKDDN model at selected time points in sunny weather.

The probabilistic prediction results of the QRKDDN model on the sunny day dataset are presented in Figure 11, while Table 2 shows the predicted values, true power values, and prediction errors for three time points. As depicted in the figure, the probability density curve is relatively full, and the observed values are located at its center, indicating that our probabilistic predictions are more reliable. Specifically, we achieved a mean absolute error of −0.199391%, 0.395191%, and 0.387813% for each of these time points, respectively, under sunny weather conditions; the overall assessment suggests that QRKDDN demonstrated superior predictive performance.

### 4.2. Cloudy

**Figure 12:**
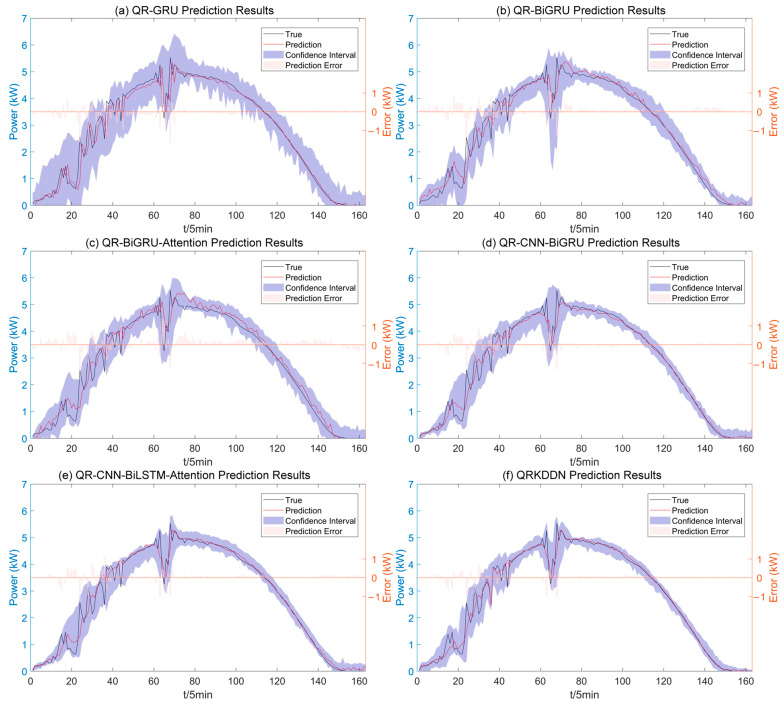
Interval prediction results for cloudy weather.

The uncertainty of weather changes is amplified under cloudy conditions, as depicted in Figure 12, which presents the interval prediction results of the QRKDDN model and the comparison model. A few predicted power points lie outside the prediction interval, primarily concentrated during periods of higher power fluctuations when wider amplitude intervals are observed. Conversely, narrower prediction intervals correspond to stable weather changes, aligning with actual weather conditions.

**Figure 13:**
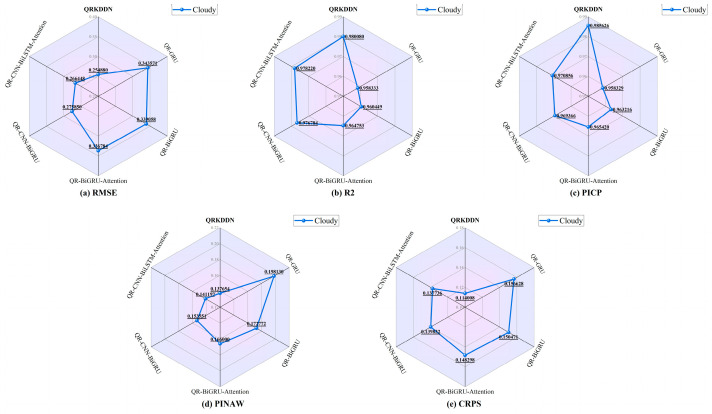
Radar chart of predictive evaluation indicators of cloudy weather.

The radar plot in Figure 13 illustrates the evaluation of prediction results for each model under cloudy weather conditions. QRKDDN consistently exhibits superior performance with a minimal PV power prediction error evaluation metric RMSE of 0.254880 and the highest goodness-of-fit $R^{2}$ value of 0.980080. Moreover, it outperforms the five comparison models in terms of interval prediction coverage, demonstrating the narrowest average width (PINAW = 0.137654) and highest coverage probability (PICP = 0.985626) for cloudy weather predictions. Additionally, QRKDDN achieves the smallest CRPS value, surpassing other models by significant margins: it is 27.21% lower than QR-GRU, 24.23% lower than QR-BiGRU, 23.12% lower than QR-BiGRU-Attention, 18.47% lower than QR-CNN-BiGRU, and finally, it is 17.23% lower than the QR-CNN-BiLSTM-Attention model.

**Figure 14:**
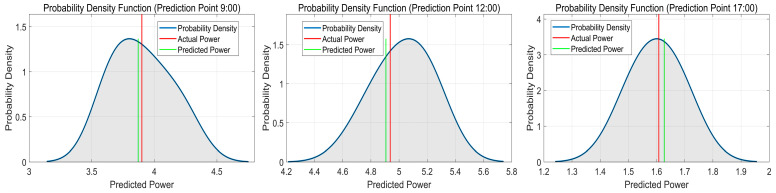
Probabilistic prediction results of QRKDDN model at selected time points in cloudy weather.

The probabilistic forecast results for cloudy weather are presented in Figure 14. Table 3 displays the predicted values, actual power values, and corresponding prediction errors at three different time points. The respective prediction errors for these time points are −0.749630%, −0.619644%, and 1.203359%.

In summary, QRKDDN exhibits the smallest deterministic prediction error, superior prediction interval coverage, and narrower interval width in cloudy weather conditions. The obtained prediction results not only meet the expected requirements, but also demonstrate the exceptional feature-mining capability of QRKDDN.

### 4.3. Rainy

**Figure 15:**
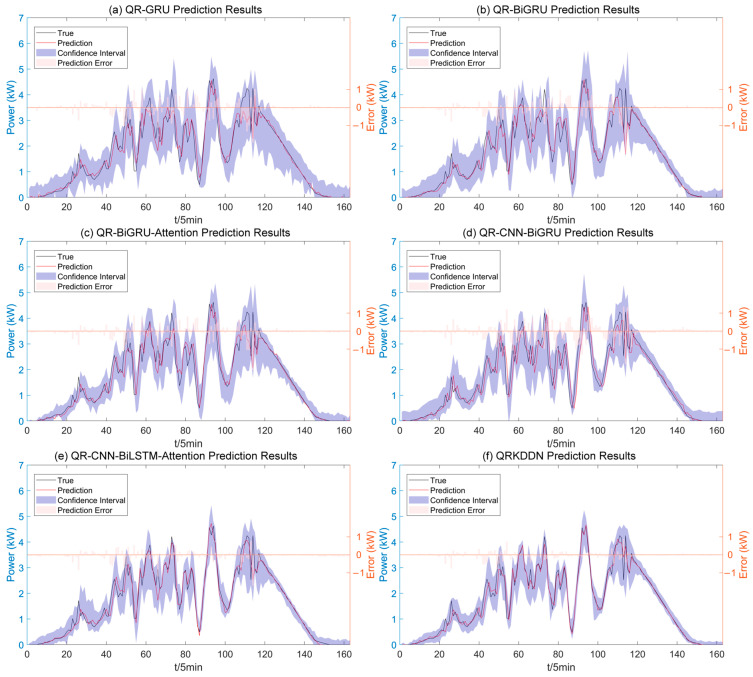
Interval prediction results for rainy weather.

In the presence of complex changes in rainy weather, PV power experiences more pronounced fluctuations. As depicted in Figure 15, the prediction results of QRKDDN and comparative models demonstrate a significant improvement in prediction error compared to sunny and cloudy weather conditions. Notably, the QRKDDN model exhibits the narrowest interval width and achieves superior interval coverage.

**Figure 16:**
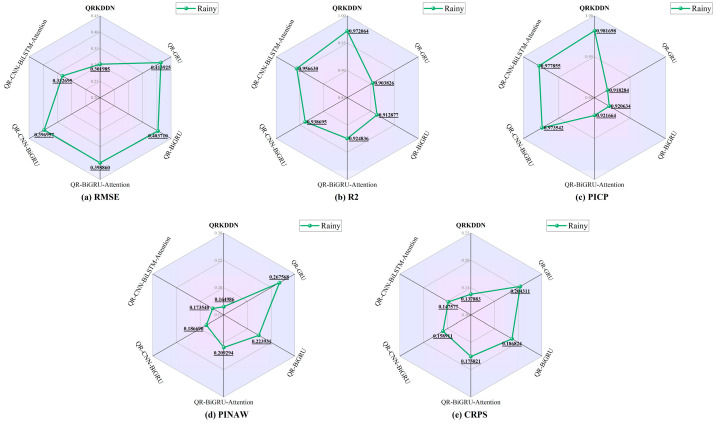
Radar chart of predictive evaluation indicators of rainy weather.

The radar charts in Figure 16 depict the evaluation of point prediction, interval prediction, and probabilistic prediction for each model under rainy weather conditions. It can be observed from the figure that QRKDDN exhibits the smallest RMSE error evaluation index value of 0.301985 and the highest goodness-of-fit $R^{2}$ value of 0.972064. Although there is a slight reduction in the accuracy of point predictions compared to sunny and cloudy weather, overall, the prediction errors meet the expected requirements satisfactorily. The prediction interval coverage reaches an impressive 98.1698%, with PINAW having the narrowest width at a value of 0.164986 among all models considered here.

Furthermore, when evaluating probabilistic predictions using CRPS as a metric, QRKDDN outperforms the other models significantly: it achieves a CRPS value that is 32.51% lower than that of the QR-GRU model, 26.20% lower than that of the QR-BiGRU model, 21.58% lower than that of the QR-BiGRU-Attention model, 13.23% lower than that of the QR-CNN-BiGRU model, and finally, it is better by being approximately 6.57% lower than the performance achieved by the QR-CNN-BiLSTM-Attention model.

The performance evaluation of time series prediction is more representative in highly fluctuating data. Therefore, we conducted a case study on rainy weather with highly fluctuating power generation to compare the effects of the CNN layer and the attention layer on the prediction model’s performance. Firstly, we compared the reference model, QR-BiGRU-Attention, with the QRKDDN prediction results. The addition of a CNN layer to QRKDDN resulted in a 24.29% lower point prediction error RMSE and a 5.11% higher $R^{2}$ compared to the QR-BiGRU-Attention model. Additionally, the interval prediction evaluation metrics showed that PICP was 6.51% higher and PINAW was 21.17% lower for QRKDDN with CNN layer integration. These findings demonstrate that CNN can effectively leverage sequence features and local details of PV power data to enhance model prediction performance.

The incorporation of an attention mechanism assigns higher weights to crucial information, thereby effectively mitigating the issue of missing data caused by long time sequences. From the prediction results, it is evident that the QRKDDN model with the attention mechanism exhibits a 23.93% reduction in point prediction error RMSE compared to the QR-CNN-BiGRU model, along with a 3.55% increase in $R^{2}$; moreover, the interval prediction evaluation indicators demonstrate a 0.84% improvement in PICP and an 11.63% decrease in PINAW, indicating that the attention mechanism significantly enhances the accuracy of time series predictions.

**Figure 17:**
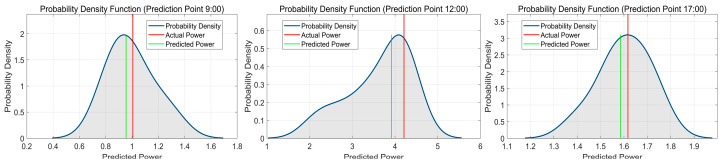
Probabilistic prediction results of QRKDDN model at selected time points in rainy weather.

The probabilistic prediction results during rainy weather are illustrated in Figure 17. Table 4 presents the predicted values, true power values, and corresponding prediction errors for the three time points. In the case of highly fluctuating rainy weather conditions, the overall probability density curves exhibit a relatively full distribution, with both true and predicted values predominantly centered within these curves. Notably, at 12:00 noon, the probability density curve indicates distinct power states or clusters near the current time point, reflecting significant power fluctuations attributed to external factors such as changes in weather patterns and cloud cover. The point prediction errors for this specific time period are −5.087916%, −6.979951%, and −1.965111%. These results demonstrate that our proposed model retains excellent predictive capabilities even under challenging circumstances characterized by drastic weather variations on rainy days; it effectively tracks PV power changes through historical meteorological data.

The prediction results demonstrate that the QRKDDN model exhibits superior overall performance in rainy weather conditions.

### 4.4. Integrated Assessment

**Figure 18:**
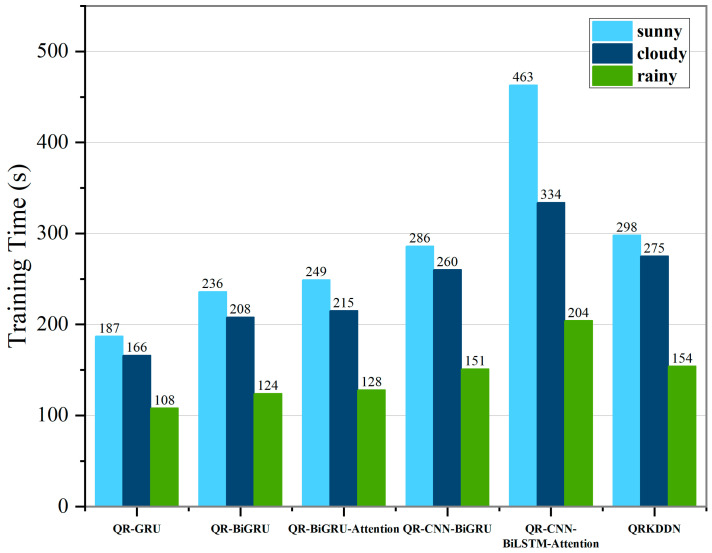
Average run time for each model.

To compare the computational efficiency of the proposed model with the comparative model, the model was configured and run 10 times according to the parameters in Table 1. The resulting average running time is illustrated in Figure 18.

The model training time was compared and analyzed using the sunny day dataset. QRKDDN incorporates an additional layer of an attention mechanism compared to QR-CNN-BiGRU, resulting in a 4.20% increase in training time; however, it leads to a certain improvement in comprehensive prediction performance. In comparison with QR-BiGRU-Attention, the inclusion of an extra CNN layer increases runtime by 19.68%, while effectively enhancing model performance, as well. Due to increased structural complexity, the training time for QRKDDN is elevated by 59.36% when compared to the QR-GRU model and by 26.27% when compared to the QR-BiGRU model; nevertheless, it experiences a reduction of 35.64% in training time relative to the QR-CNN-BiLSTM-Attention model. These results demonstrate that although there is a delay in running speed for QRKDDN compared to single structure models like GRU, its prediction accuracy is significantly improved; moreover, the excellent architecture of QRKDDN substantially reduces training time when contrasted with the QR-CNN-BiLSTM-Attention model.

**Figure 19:**
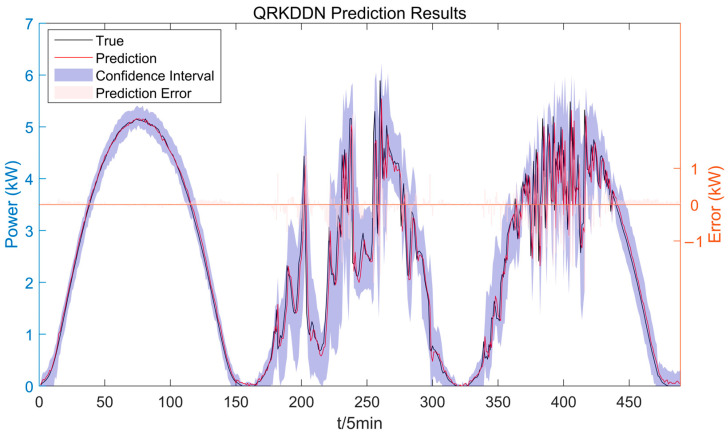
QRKDDN unsimilar day clustering interval prediction results.

To comprehensively compare the enhancement effect of GMM similar day clustering, Figure 19 presents the prediction results and evaluation index cube diagrams of QRKDDN without employing similar day clustering. Specifically, we selected a period of three days (from 5 November to 7 November) with typicality for display purposes. The ratio of the training set and test set was 7:3, which was consistent with the clustering prediction.

The results presented in Table 5 demonstrate that the QRKDDN model, when not incorporating the GMM clustering algorithm, exhibits significantly larger errors in both point prediction and interval prediction compared to those obtained from similar day clustering prediction results. This observation highlights the superiority of the GMM similar day clustering algorithm in PV power prediction.

To demonstrate the enhanced prediction performance of QRKDDN compared to traditional models, this paper selects classical models such as LSTM, CNN, RNN, and ELM (extreme learning machine) for comparison. Each model was validated 10 times using the rainy dataset as an example, and the prediction results are presented in Table 6. As shown in Table 6, QRKDDN exhibits a longer computational time compared to other methods due to its complex network structure. However, when compared with methods like RNN, the difference in training time is not significant and still meets practical application requirements. The evaluation metrics for point prediction and probabilistic prediction indicate that QRKDDN outperforms traditional models by accurately predicting PV power generation. This provides robust data support for decision makers in power system management.

## 5. Conclusions

The present study proposes a QRKDDN PV power interval probabilistic prediction model. Firstly, meteorological variables highly correlated with PV power were selected using the Pearson correlation coefficient method. Secondly, a multivariate multi-feature-based GMM clustering algorithm was employed to cluster the historical data. Finally, the time series prediction performance of QRKDDN was validated on similar daily datasets representing three weather types: sunny, cloudy, and rainy. For performance comparison purposes, the QR-GRU, QR-BiGRU, QR-BiGRU-Attention, QR-CNN-BiGRU, and QR-CNN-BiLSTM-Attention models were chosen as benchmark models. The results demonstrate that the interval prediction performance of the proposed QRKDDN model surpasses that of the other models due to its well-designed structure, which effectively captures deeper features among variables during drastic weather changes. This reduction in prediction uncertainty enables reliable probabilistic predictions for decision making in power system operation and maintenance.

The utilization of a data-driven approach derived from sensors plays an indispensable role in enhancing the precision and comprehensiveness of contemporary time series prediction research. Due to the limited availability of data resources, the proposed method was solely validated using photovoltaic power data. Meanwhile, the proposed QRKDDN model still exhibits the characteristics of high model complexity, relatively long running time, and demanding hardware requirements. Therefore, we intend to further explore efficient optimization algorithms to enhance the performance of the model. Furthermore, we aim to enhance the applicability of this approach by conducting practical validation in diverse domains, such as wind power forecasting, power load estimation, and battery life prediction, in future investigations.

## Figures and Tables

**Table 1:** Comparative analysis of model structural parameter settings and performance.

Models	Parameters	Characteristic	Possible Defects
QR-GRU [43]	Number of GRU units: 128	Simple model structure and fast running speed	Inadequate capacity to capture long-term dependencies in time series data
QR-BiGRU [40]	Number of BiGRU units: 128	Effectively capturing bidirectional dependencies in sequential data	Overfitting may arise in certain elementary sequences.
QR-BiGRU-Attention [44]	BiGRU layer: 128 BiGRU units Attention layer: assigned according to weights	The attention mechanism effectively enhances the model’s focus on crucial information.	Prone to interference from noise in sequence data
QR-CNN-BiGRU [45]	CNN_1 layer: 64 convolutional kernels, Kernel size: 4 Padding: same CNN_2 layer: 128 convolutional kernels, Kernel size: 4, Padding: same BiGRU layer: 128 BiGRU units Activation function: ReLU	Efficiently integrating local and global information in time series analysis	The key model information cannot be captured accurately.
QR-CNN-BiLSTM-Attention [46]	CNN_1 layer: 64 convolutional kernels CNN_2 layer: 128 convolutional kernels BiLSTM layer: 128 BiLSTM units Activation function: ReLU	Reconciling the strengths of each model in a synergistic manner to transcend the limitations inherent in any single model	The extended duration of operation and relatively limited capability in extracting features
QRKDDN	CNN_Layer 1: 64 convolutional cores Kernel size: 4 Padding: same Activation function: ReLU CNN_Layer 2: 128 convolutional cores Kernel size: 4 Padding: same Activation function: ReLU BiGRU layer: 128 BiGRU units MaxPooling: Pooling size: 3 Step length: 2 Attention layer: assigned according to weights	The model exhibits exceptional predictive performance, demonstrates a robust ability to capture features during temporal changes, and offers relatively efficient time series prediction.	The complexity of the training process is high and may necessitate greater computational resources.
Sliding window width		18	
Forecast time step		1	
Training rounds		200	
Batch size		128	
Dropout		0.2	
Initial learning rate		0.01	
Learning rate decay factor		0.1	
Minimum learning rate		0.001	
Training/test set ratio		0.7/0.3	
Cross-validation method		Rolling cross validation	
Loss function		MSE	
Optimizer		Adam	

**Table 2:** Prediction error of QRKDDN model for selected time points in sunny weather.

	9:00	12:00	17:00
Predicted Value (kW)	3.233906	5.067883	1.678685
Actual Value (kW)	3.240367	5.047934	1.672200
Prediction Error (%)	−0.199391	0.395191	0.387813

**Table 3:** Prediction error of QRKDDN model for selected time points in cloudy weather.

	9:00	12:00	17:00
Predicted Value (kW)	3.871129	4.906641	1.627384
Actual Value (kW)	3.900367	4.937234	1.608033
Prediction Error (%)	−0.749622	−0.619638	1.203396

**Table 4:** Prediction error of QRKDDN model for selected time points in rainy weather.

	9:00	12:00	17:00
Predicted Value (kW)	0.955164	3.916206	1.585224
Actual Value (kW)	1.006367	4.210067	1.617000
Prediction Error (%)	−5.087905	−6.979960	−1.965121

**Table 5:** Predicted evaluation indicators.

Dataset	RMSE	R^2^	PICP	PINAW
Sunny (Clustered)	0.029120	0.999869	1.000000	0.035062
Cloudy (Clustered)	0.254880	0.980080	0.985626	0.137654
Rainy (Clustered)	0.301985	0.972064	0.981698	0.164986
Weather Unclustered	0.393528	0.960222	0.953765	0.181110

**Table 6:** Predicted results and training time (QRKDDN vs. baseline model).

Method	Model Training Time (Average of 10 Times)	Rainy			
		RMSE	R^2^	PICP(%)	PINAW
QR-LSTM [29]	118	0.874331	0.857201	0.883229	0.223071
QR-CNN [11]	71	0.624928	0.874490	0.902856	0.207942
QR-RNN [28]	137	0.562490	0.929510	0.918628	0.196856
QR-ELM [11]	53	1.085463	0.825938	0.865367	0.246330
QRKDDN	154	0.301985	0.972064	0.981698	0.164986

## Data Availability

The required datasets for the experiment can be obtained for free from https://dkasolarcentre.com.au/ (accessed on 4 May 2023).
